# Role of Mitofusins and Mitophagy in Life or Death Decisions

**DOI:** 10.3389/fcell.2020.572182

**Published:** 2020-09-22

**Authors:** Mariana Joaquim, Mafalda Escobar-Henriques

**Affiliations:** Institute for Genetics, Cologne Excellence Cluster on Cellular Stress Responses in Aging-Associated Diseases (CECAD), Center for Molecular Medicine Cologne (CMMC), University of Cologne, Cologne, Germany

**Keywords:** mitochondria, mitofusins, MFN2, mitophagy, apoptosis, NAFLD

## Abstract

Mitochondria entail an incredible dynamism in their morphology, impacting death signaling and selective elimination of the damaged organelles. In turn, by recycling the superfluous or malfunctioning mitochondria, mostly prevalent during aging, mitophagy contributes to maintain a healthy mitochondrial network. Mitofusins locate at the outer mitochondrial membrane and control the plastic behavior of mitochondria, by mediating fusion events. Besides deciding on mitochondrial interconnectivity, mitofusin 2 regulates physical contacts between mitochondria and the endoplasmic reticulum, but also serves as a decisive docking platform for mitophagy and apoptosis effectors. Thus, mitofusins integrate multiple bidirectional inputs from and into mitochondria and ensure proper energetic and metabolic cellular performance. Here, we review the role of mitofusins and mitophagy at the cross-road between life and apoptotic death decisions. Furthermore, we highlight the impact of this interplay on disease, focusing on how mitofusin 2 and mitophagy affect non-alcoholic fatty liver disease.

## Introduction

**FIGURE 1:**
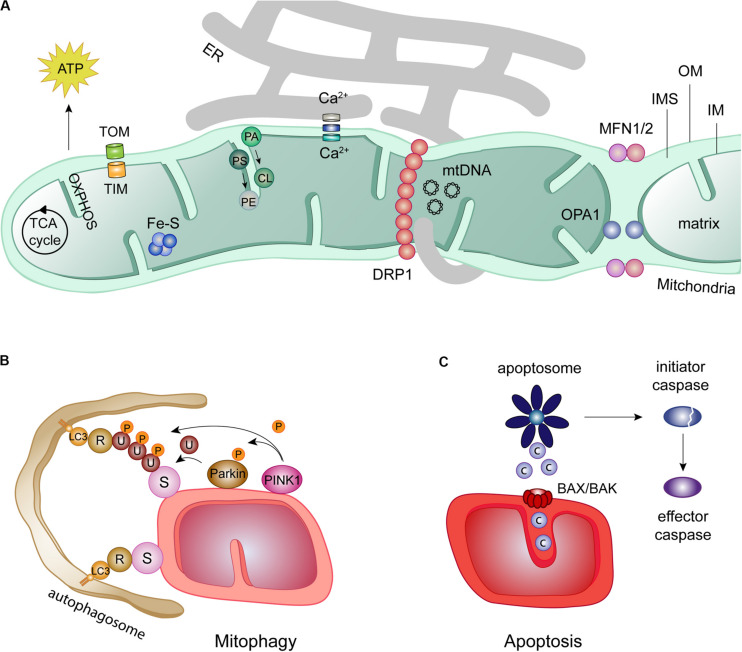
Mitochondrial roles in health and disease. **(A)** Despite the presence of an own DNA in the mitochondrial matrix (mtDNA), most mitochondrial proteins are imported from the cytosol by two translocase complexes: the translocase of the outer membrane (TOM) and the translocase of the inner membrane (TIM). Mitochondria are hubs for several cellular processes, such as iron-sulfur clusters (Fe-S) assembly, metabolite oxidation by the tricarboxylic acid (TCA) cycle and ATP production via the oxidative phosphorylation chain (OXPHOS). Further, mitochondrial proximity to the endoplasmic reticulum (ER) regulates calcium (Ca^2+^) buffering and phospholipid synthesis, e.g., cardiolipin (CL) and phosphatidylethanolamine (PE) from their ER precursors phosphatidic acid (PA), and phosphatidylserine (PS), respectively. ER-mitochondrial contacts also regulate mitochondrial fission, by facilitating the recruitment of DRP1 to the mitochondria. In turn, mitochondrial fusion requires the mitofusins MFN1 and MFN2 at the OM and OPA1 at the IM. **(B)** Mitochondrial function is kept in check by mitophagy, a quality control mechanism. Mitophagy can occur dependently or independently of ubiquitin. The canonical ubiquitin-dependent PINK1/Parkin pathway initiates with the accumulation of the kinase PINK1 at the OM, which recruits the E3 ligase Parkin. Ubiquitylation (U) of several OMM substrates (S) by Parkin and additional phosphorylation (P) of ubiquitin and Parkin generates a positive feedback loop increasing Parkin activity. The ubiquitin chains formed on OM substrates bind to the lipidated autophagosome receptor LC3, via receptors proteins (R). Mitochondria are then surrounded and engulfed by the autophagosome, which finally fuses with the lysosome for degradation. The ubiquitin-independent mitophagy only requires the recognition of OM substrates (S) by lipidated LC3 directly via mitochondrial receptors (R). **(C)** Mitochondria are directly involved in the initiation of apoptosis via the intrinsic apoptotic pathway. In this pathway, intrinsic death stimuli induce permeabilization of the OM by oligomerization of the pro-apoptotic BCL-2 proteins BAX and BAK. Apoptotic molecules such as cytochrome c (c) are release form the IMS, activating the apoptotic complex apoptosome. This complex is able to cleaved and thereby activate initiator caspases which, in turn, activate effector caspases.

Mitochondrial biology has raised extensive research interest, thanks to its expanding roles in tailored metabolic performance, in quality control responses, but also in inflammatory processes and in cell death (Westermann, 2010; Nunnari and Suomalainen, 2012; Xiong et al., 2014; Angajala et al., 2018; Pickles et al., 2018; Spinelli and Haigis, 2018; Figure 1). Mitochondria are double membrane organelles, being its structure and biogenesis extensively described (Pfanner et al., 2019). The outer membrane (OM) provides the first semi-permeable barrier to the cytoplasm. It contains the protein and lipid receptors of mitophagy and is a critical determinant of cell death triggers (Harper et al., 2018; Pickles et al., 2018; Xie et al., 2018; Sedlackova and Korolchuk, 2019). The OM also anchors the effectors of mitochondrial intraorganellar fusion and protein complexes forming interorganellar contact sites. These sustain, for example, mitochondria and endoplasmic reticulum (ER) exchanges, which are determinant for calcium (Ca^2++^) buffering and phospholipid transfer (Marchi et al., 2017; Figure 1A). The inner membrane (IM), thanks to its impermeability, maintains the proton motive force necessary for mitochondrial biogenesis and energy conversion. By folding on itself, the IM creates invaginations called cristae, where the mitochondrial oxidative phosphorylation system (OXPHOS) is located (Frey et al., 2002). Their electron shuttling and proton pumping capacity sustains the mitochondrial membrane potential, enabling the production of energy (Zhao et al., 2019). In addition to ATP production, the IM is also in charge of phospholipid synthesis (Tatsuta and Langer, 2017). The different cristae are spaced by IM portions lining parallel to the OM, called inner boundary membrane, being the connection points defined as cristae junctions. The inter membrane space (IMS)–the small aqueous compartment confined between both mitochondrial membrane-s is a signaling hub reservoir, in both pro-survival and cell death responses. It allows the accumulation of the protons released by the electron transport chain and comprises cytochrome c (CytC) the high-temperature requirement protein A2 (Htr2/Omi), the apoptosis inducing factor (AIF), the second-mitochondria-derived activator of caspases (Smac), the direct IAP-binding protein with low PI (Diablo) and Endonuclease G, which differently support mitochondrial metabolism (Herrmann and Riemer, 2010). However, once released from the mitochondria into the cytoplasm, upon cristae opening, these pleiotropic proteins convert into apoptosis initiators, pushing the cell toward a deadly end (Snigirevskaya and Komissarchik, 2019). Besides apoptosis, mitochondria are also recognized by regulating other types of cell death such as necroptosis, ferroptosis, pyroptosis, and mitochondrial-mediated necrosis (Baines, 2010; Baker et al., 2014; Marshall and Baines, 2014; Battaglia et al., 2020; Kai et al., 2020; Wang et al., 2020; Wei et al., 2020). Finally, the IM encloses the mitochondrial matrix, where essential reactions take place, such as iron-sulfur cluster assembly (Cardenas et al., 2018; Lill, 2020) or the tricarboxylic acid (TCA) cycle, which feeds electrons to the respiratory chain and provides amino acid precursors (Mailloux et al., 2007; Martínez-Reyes and Chandel, 2020). Finally, the matrix harbors the mitochondrial DNA and respective transcription and translation machineries (Mazunin et al., 2015; Figure 1A).

Mitochondria are constantly reshaped, by fusion and fission events of the whole organelle, but also by alterations in cristae organization, altering access of the IMS content to the OM (Rampelt et al., 2017; Giacomello et al., 2020). Mitochondrial adaptive morphology, through the shift in activity of its fission and fusion machineries, is essential for their correct functioning (Chan, 2012; Buck et al., 2016; Cantó, 2018; Tilokani et al., 2018; Dorn, 2019; Whitley et al., 2019). It capacitates mitochondria to respond to cellular cues both in healthy and stress situations (Liesa and Shirihai, 2013; Mishra and Chan, 2016; Schrepfer and Scorrano, 2016; Chen and Chan, 2017; Eisner et al., 2018; Zemirli et al., 2018). While, for example, nutrient starvation shifts the balance toward a tubular mitochondrial network (Tondera et al., 2009; Gomes et al., 2011; Rambold et al., 2011), loss of membrane potential and nutrient excess were shown to induce mitochondrial fragmentation (Yu et al., 2006). The mitochondrial morphology machinery is composed by DRP1 (Dnm1 in yeast), responsible for fission, Mitofusin 1 and Mitofusin 2 (MFN1/MFN2) (Fzo1 in yeast), responsible for OM fusion and OPA1 (Mgm1 in yeast), responsible for IM fusion (Youle and van der Bliek, 2012; Tilokani et al., 2018; Figure 1A). Recent developments highlighted novel molecular determinants of mitochondrial ultrastructure dynamics (Xie et al., 2018; Kondadi et al., 2019; Rastogi et al., 2019; Snigirevskaya and Komissarchik, 2019). The fascinating plasticity of mitochondrial morphology is also brought about by post-translational modifications of the fusion and fission components, including ubiquitylation, phosphorylation, sumoylation, and proteolytic processing (Escobar-Henriques and Langer, 2014; Hofer and Wenz, 2014; Macvicar and Langer, 2016; Mishra and Chan, 2016). The mitofusins MFN1 and MFN2 are ubiquitylated by different E3 ligases, in response to a big variety of stimuli, which tightly regulate their fusion properties, mitochondria-ER contact sites, mitophagy and apoptosis (Escobar-Henriques and Joaquim, 2019).

In sum, mitochondria are docking stations for cellular fitness (Abate et al., 2019). By integrating external signals, which change their metabolism, shape and signaling response properties, mitochondria dictate life and death decisions. In turn, by sequestering pro-apoptotic molecules that are only to be released in the presence of death stimuli, mitochondria themselves must be tightly regulated, to prevent undesired cell death. Thus, elimination of dysfunctional mitochondria by mitophagy is critical for cellular survival (Pickles et al., 2018; Allen and Baehrecke, 2020; Markaki and Tavernarakis, 2020). Despite being mostly pro-survival, mitophagy can also synergize with apoptosis to instead promote cell death (Yee et al., 2010; Panda et al., 2018; Ding et al., 2019; Han et al., 2019). Here, we discuss how the mitochondrial fusion factors, mitofusins, impact on mitophagy and apoptosis, present pro-survival and pro-apoptotic roles of mitophagy and detail the roles of mitophagy and mitofusin 2 in non-alcoholic fatty liver disease (NAFLD).

## The Mitofusin Proteins, MFN1 and MFN2

Mitofusin 1 and Mitofusin 2 are homologous conserved transmembrane proteins, being mainly exposed to the cytosol (Zorzano and Pich, 2006). They possess a GTPase domain at the N-terminal and two hydrophobic heptad repeat domains, separated by transmembrane anchor(s) (Hales and Fuller, 1997; Rojo et al., 2002; Low et al., 2009; Qi et al., 2016; Cao et al., 2017; Daste et al., 2018; Mattie et al., 2018; Yan L. et al., 2018; Li et al., 2019). Despite having 77% of similarity, their deletion differentially affects mitochondrial morphology (Chen et al., 2003). While depletion of *MFN1* leads to highly fragmented mitochondria, organized in small fragments dispersed throughout the cytosol, depletion of its homolog *MFN2* leads to bigger mitochondrial fragments that cluster perinuclearly. However, overexpression of either Mfn1 or Mfn2 in single and double mitofusins knockout murine fibroblasts leads to complete rescue of the mitochondrial morphology phenotypes (Detmer and Chan, 2007). Although ubiquitously expressed, *MFN1* is mainly present in heart and testis, while *MFN2* is predominant in the brain and muscle tissues; in other tissues, mitofusins present similar expression levels (Eura et al., 2003; Santel et al., 2003). Murine homozygous deletion of *Mfn1* or *Mfn2* is lethal and double-knockout mice die even earlier than single knockouts (Chen et al., 2003), suggesting that they play non-redundant roles. Additionally, mice depleted for *Mfn2* (but not for *Mfn1*) present placental defects within the giant cell layer (Chen et al., 2007). If *Mfn2* is only depleted after placentation, it leads to cerebellar neurodegeneration (Chen et al., 2007), highlighting different impact of these proteins according to the developmental stage.

Although both mitofusins regulate mitochondrial fusion, additional roles have been attributed to MFN2 (Chen and Chan, 2017; Filadi et al., 2018; Mattie et al., 2019; Dorn, 2020). MFN2 was proposed to regulate tethering and distance between mitochondria and ER (De Brito and Scorrano, 2008; Cosson et al., 2012; Filadi et al., 2015; Naon et al., 2016), controlling the Ca^2+^ exchange between both organelles (Rizzuto et al., 1998; Szabadkai et al., 2006). Consistently, Mfn2 and ER-mitochondrial contacts modulated Ca^2+^-dependent roles in vascular remodeling (Zhu et al., 2017; Göbel et al., 2020). Moreover, mitochondria-ER contact sites are essential for phospholipid transfer between the two organelles (Kojima et al., 2016), being a direct role of MFN2 in lipid transfer recently proposed (Hernández-Alvarez et al., 2019). However, it is still under debate under which conditions Mfn2 acts as a spacer between both organelles, consistent with decreased distance observed in its absence (Cosson et al., 2012; Filadi et al., 2015; Wang et al., 2015) or rather acts as an ER-mitochondria tether (De Brito and Scorrano, 2008; Naon et al., 2016; Basso et al., 2018; McLelland et al., 2018). MFN2 has also been extensively linked to mitophagy (Gegg et al., 2010; Poole et al., 2010; Ziviani et al., 2010), to apoptosis (Karbowski et al., 2002; Brooks et al., 2007; Hoppins et al., 2011), as detailed below, and recently also to pyroptosis (Kai et al., 2020) and ferroptosis (Wei et al., 2020). Furthermore, MFN2 is believed to play multiple roles in metabolism, explaining its involvement in metabolic disorders such as obesity and diabetes mellitus (Bach et al., 2005; Toledo et al., 2006; Sebastián et al., 2012; Schneeberger et al., 2014; Boutant et al., 2017; Ramiréz et al., 2017; Bell et al., 2019). *MFN2* depletion leads to reduced mitochondrial membrane potential, oxygen consumption rate and mitochondrial proton leakage and impairs glucose, pyruvate and fatty acid oxidation (Bach et al., 2003; Chen et al., 2005; Pich et al., 2005; Mourier et al., 2015). Importantly, *Mfn2* loss-of-function represses nuclear-encoded subunits of OXPHOS complexes I, II, III and V (Pich et al., 2005), independently of its fusogenic role (Pich et al., 2005; Loiseau et al., 2007). Moreover, mitofusins were shown to be required for mitochondrial DNA (mtDNA) replication and nucleoid distribution (Chen et al., 2010; Ramos et al., 2019). Both mitofusins have also been associated with female fertility (Liu et al., 2016; Zhang et al., 2019a,b). Mfn2 is important for oocyte and follicle development (Liu et al., 2016) and both mitofusins are required for the maintenance of the ovarian follicular reserve (Zhang et al., 2019a,b). Regarding cell cycle progression, *MFN2* overexpression supressed cellular proliferation (Cheng et al., 2013) and MFN2 depletion increased it, dependent on the Ras-Raf-ERK signaling pathway (Chen et al., 2014). The role of mitofusin 2 in controlling cell proliferation possibly explains its link with cancer. Indeed, *MFN2* overexpression is able to slow the growth of different cancer cell lines (Xie et al., 2015; Xu et al., 2017). Furthermore and quite controversially, lack of *MFN2* seems to equally impair both stem cell self-renewal and differentiation capacity (Kasahara et al., 2013; Fang et al., 2016; Khacho et al., 2016).

Mitofusin 1 and Mitofusin 2 have a clearly different biological impact, perhaps explaining the inexistence of MFN1 mutations causing human diseases. In contrast, more than a hundred *MFN2* mutations are known to cause the Charcot-Marie-Tooth Type 2A (CMT2A) disorder (Stojkovic, 2016; Dohrn et al., 2017), a subtype of the incurable peripheral neuropathy Charcot-Marie-Tooth (CMT). CMT affects about 1 in 2500 people, being the most common inherited neurological disease and is characterized by progressive distal weakness, muscular atrophy, and sensory abnormalities (Tazir et al., 2013; El-abassi et al., 2014; Stuppia et al., 2015; Stojkovic, 2016; Barbullushi et al., 2019). The restoration of mitochondrial fusion by either transgenic overexpression of Mfn1 or by Mfn2 agonist molecules in murine models led to reversion of some of the CMT2A defects (Rocha et al., 2018; Zhou Y. et al., 2019). However, to date, the disease-underlying functions of MFN2 in CMT2A remain elusive. So far, reports have pointed to apoptosis resistance and increased mitophagy, observed in iPSCs-derived CMT2A motor neurons lines (Rizzo et al., 2016). Importantly, different CMT2A disease mutant cell lines have displayed impaired ER-mitochondria contacts, as well as ER stress, defective Ca^2+^ uptake and phospholipid synthesis and transfer (Bernard-Marissal et al., 2018; Larrea et al., 2019), pointing to a possible role of these contact sites at the basis of CMT2A disease. Besides CMT2A, MFN2 has been linked to a variety of diseases (Chandhok et al., 2018; Filadi et al., 2018). The most described links are with prevalent neuropathies such as Parkinson’s and Alzheimer’s disease (Han et al., 2011; Lee et al., 2012; Stuppia et al., 2015; Gao et al., 2017), cardiac dysfunction (Hall et al., 2014; Nan et al., 2017; Dorn, 2018; Hernandez-Resendiz et al., 2020), type 2 diabetes, obesity and insulin resistance (Zorzano et al., 2009; Dai and Jiang, 2019) and cancer (Allegra et al., 2019). Finally, MFN2 has also been associated with progression of liver diseases such as acute-on-chronic liver failure (ACLF) and NAFLD (Wang et al., 2013; Hernández-Alvarez et al., 2019; Xue et al., 2019a,b) and proposed as a possible therapeutic target for hepatic inflammation and fibrosis (Zhu et al., 2020).

## Regulation of Mitophagy by Mitofusins

Mitochondrial homeostasis is ensured by the coordination between its biogenesis rate, enabling the replenishment of novel healthy organelles, and the elimination of the superfluous or damaged mitochondria by selective self-digestion, via mitophagy (Harper et al., 2018; Pickles et al., 2018; Pfanner et al., 2019; Allen and Baehrecke, 2020; Markaki and Tavernarakis, 2020). Mitophagy requires the recognition of mitochondrial adaptors by receptors on the autophagosome, the double membrane autophagic vacuole responsible for engulfment of the material to be degraded (Lahiri et al., 2019; Allen and Baehrecke, 2020; Figure 1B). Moreover, mitochondrial elimination requires the ubiquitin-like modifier LC3 (Atg8), whose lipidated and active form integrates into the autophagosome membrane. LC3 is recognized by specific receptors, either present at the mitochondrial OM, like Atg32, NIX and BNIP3, or instead soluble at the cytoplasm and being recruited to the OM, like Optineurin, NDP52, p62, NBR1, and TAX1BP1 (Geisler et al., 2010; Narendra et al., 2010; Lazarou et al., 2015; Khaminets et al., 2016; Mcwilliams and Muqit, 2017). In fact, these receptors interact with both ubiquitin and LC3 interacting (LIR) motifs, being therefore recruited to mitochondria by ubiquitylated OM proteins. Once loaded with damaged mitochondria, the autophagosome then fuses with the lysosome, forming the autolysosome, where mitochondrial degradation takes place (Allen and Baehrecke, 2020). Upon acute stress conditions, fission and selective fusion were shown to facilitate segregation and subsequent turnover of the damaged pieces (Twig et al., 2008; Burman et al., 2017). Mitofusins have been extensively implicated in mitochondrial quality control, mainly attributed to their decisive role in mitochondrial length and their receptor property for mitophagy effectors (Dorn, 2020).

The general ubiquitylation of OM proteins is one of the early steps and a hallmark in mitophagy (Palikaras et al., 2018; Wang Y. et al., 2019; Figure 1B). The most extensively studied ubiquitin-dependent pathway is undertaken by the serine/threonine kinase PINK1 and by Parkin, a RING-between-RING E3 ubiquitin ligase (Pickles et al., 2018). Under healthy conditions, PINK1 is inactivated by proteasomal turnover. First, PINK1 is imported through the TOM and TIM23 translocator complexes (Lazarou et al., 2012; Sim et al., 2012; Sekine and Youle, 2018). PINK1 is then constitutively cleaved by the IM residing protease PARL, being its truncated soluble form extracted back to the cytosol and degraded by the proteasome (Yamano and Youle, 2013). However, upon mitochondrial stress, decreased mitochondrial membrane potential prevents PINK1 import, consequently accumulating its full-length form at the OM, exposing its kinase domain to the cytosol (Matsuda et al., 2010; Vives-Bauza et al., 2010). Mitophagy is then initiated by phosphorylation of ubiquitin molecules at serine 65 (Kane et al., 2014; Koyano et al., 2014) and of Parkin (Shiba-Fukushima et al., 2012; Figure 1A). This shifts Parkin to an active conformation, fostering its recruitment to mitochondria and potentiating ubiquitylation of OM proteins (Gegg et al., 2010; Poole et al., 2010; Tanaka et al., 2010; Ziviani et al., 2010; Chan et al., 2011; Glauser et al., 2011; Rakovic et al., 2011; Riley et al., 2013; Spratt et al., 2013; Wauer and Komander, 2013; Caulfield et al., 2014; Kane et al., 2014; Kazlauskaite et al., 2014; Koyano et al., 2014; Swatek and Komander, 2016; Wauer et al., 2016; Kumar et al., 2017; Mcwilliams et al., 2018; Gladkova et al., 2019). The continuous phosphorylation of the poly-ubiquitin chains by PINK1 creates a positive feed-forward cycle, which massively increases Parkin recruitment to mitochondria and ubiquitylation of OM proteins (Ordureau et al., 2014). Besides Parkin, other E3 ligases have been shown to ubiquitylate OM proteins and thus signal mitophagy, like MARCH5, Gp78 and MGRN1, HUWE1, MUL1, and SIAH-1 (Liu et al., 2012; Fu et al., 2013; Cilenti et al., 2014; Yun et al., 2014; Tang et al., 2015; Mukherjee and Chakrabarti, 2016a,b; Szargel et al., 2016; Chen et al., 2017; Daskalaki et al., 2018; Di Rita et al., 2018; Ferrucci et al., 2018; Lampert et al., 2019; Shefa et al., 2019).

Mitofusins are preferred targets at the OM, being ubiquitylated by all above-mentioned E3 ligases (extensively reviewed in Escobar-Henriques and Joaquim, 2019). They are among the first substrates to be ubiquitylated by Parkin, mostly observed upon Parkin overexpression (Chan et al., 2011; Sarraf et al., 2013; McLelland et al., 2018) and therefore possibly reflecting experimental artifacts. Nevertheless, the ubiquitylation of mitofusins by Parkin was equally demonstrated to occur and be important for mitophagy in the absence of overexpression, with endogenous PINK1 and Parkin, in reprogrammed induced neuron cells (Ordureau et al., 2020). Upon mitophagy induction, ubiquitylation of mitofusins 1 and 2 targets them for degradation by the proteasome, quickly leading to abrogation of mitochondrial fusion events, resulting in mitochondrial fragmentation (Geisler et al., 2010; Narendra et al., 2010; Lazarou et al., 2015; Khaminets et al., 2016; Mcwilliams and Muqit, 2017). However, in the above-mentioned induced neurons -a form of not fully differentiated immature neuronal cells converted from human embryonic stem cells- extraction from the OM and proteasomal degradation of mitofusins was not required for mitophagy (Ordureau et al., 2020). This favors the previously suggested pro-mitophagy role of mitofusins as autophagic receptors (Chen and Dorn, 2013; Song et al., 2015). Loss of *MFN2*/*Mfn2* has also been connected with a decrease in autophagosome formation and/or defects in autophagosome-lysosome fusion, two events of mandatory nature for mitophagy to occur (Zhao et al., 2012; Sebastián et al., 2016; Peng et al., 2018). Consistently, depletion of both *Mfn1* and *Mfn2* in murine cardiomyocytes caused accumulation of defective mitochondria (Song et al., 2014, 2015). Equally supporting a pro-mitophagic role of mitofusins, knockdown of *Mfn1* led to mitophagy inhibition caused by overexpression of the E3-ligase Gp78 (Fu et al., 2013). In contrast, an active role of MFN2/Mfn2 in preventing mitophagy was also proposed, connected to the ER-mitochondrial tether function of MFN2 (Basso et al., 2018; McLelland et al., 2018). Consistently, CMT2A-linked *MFN2* mutants caused increased autophagic flux, whereas MFN2 overexpression prevented autophagy (Rizzo et al., 2016; Ying et al., 2017). Finally, as detailled bellow, upon peripheral nerve injury *Mfn1* depletion induced mitophagy and apoptosis (Yang et al., 2020). Finaly, mitophagy progression was even proposed not to depend on mitofusins (Narendra et al., 2008; Chan et al., 2011). In brief, how mitofusins affect mitophagy might be context-dependent, which is perhaps not so surprising considering the multi-functionality of MFN2 and its responsiveness to many different stress conditions.

## Mitofusins and Apoptosis

Cell death by apoptosis is an essential mechanism for cellular turnover, which occurs via a programmed and tightly regulated way (Kerr et al., 1972; Adams and Cory, 2007; Nair et al., 2014). It is important in physiological conditions, e.g., during embryonic development or neuronal network formation, but also under pathological conditions, e.g., for tissue homeostasis in response to stress (Elmore, 2007). Apoptosis can be induced via two distinct pathways, extrinsic or intrinsic, culminating in the activation of different cysteine-dependent aspartate-directed proteases (caspases), the final effectors of apoptotic cell death (Meier and Vousden, 2007). The extrinsic apoptotic pathway is initiated by external cellular signals, followed by binding of a death ligand to a cell-surface receptor (Jin and El-Deiry, 2005; Nair et al., 2014). The intrinsic apoptotic pathway, known as mitochondrial apoptotic pathway, is initiated by the release of AIFs from the IMS to the cytosol (Bock and Tait, 2020), such as CytC, SMAC/DIABLO and HtrA2/Omi (Griffiths et al., 1999; Antonsson et al., 2001; Wang and Youle, 2009; Figure 1C). This requires mitochondrial OM permeabilization (MOMP) (Edlich et al., 2011; Todt et al., 2015). MOMP is mediated by oligomers of BAX and BAK, the two main regulators of mitochondrial apoptosis, which in the absence of stress are constantly translocated between the cytosol and mitochondria (Todt et al., 2015). Upon death stimuli, BAX and BAK accumulate at the mitochondrial OM, bind to death signal sensors (BH3 domain-only proteins), undergo conformational changes and oligomerize, enabling MOMP. Once in the cytosol, CytC binds to the apoptotic protease activating factor-1 (APAF-1), activating its nucleotide exchange factor activity and forming a homoheptameric APAF-1 complex, named apoptosome (Liu et al., 1996; Li et al., 1997). Finally, the apoptosome cleaves and activates pro-caspase-9, necessary for activation of downstream effector caspases (Xiong et al., 2014). In general lines, effector caspases induce endonucleases and proteases to degrade nuclear material and key structural proteins (Hengartner, 2000; Slee et al., 2001). The action of the effector caspases over their targets leads to apoptotic morphological traces, such as cytoplasmic disorganization, cell shrinkage, chromatin condensation, DNA fragmentation and apoptotic body formation.

The key proteins mediating mitochondrial shape–OPA1, mitofusins and DRP1- were also implicated in the regulation of apoptosis (Campello and Scorrano, 2010; Karbowski, 2010; Xie et al., 2018). The cristae remodeling events necessary for release of CytC from cristae junctions were proposed to be mediated by OPA1 (Scorrano et al., 2002; Cipolat et al., 2006; Frezza et al., 2006). In turn, DRP1 and mitofusins have been shown to interact with BAX and BAK (Karbowski et al., 2002, 2006; Brooks et al., 2007). Upon apoptosis induction, activated BAX is recruited to MFN2-containing puncta (Neuspiel et al., 2005), suggesting a synergistic relation between MFN2 and pro-apoptotic proteins. Indeed, overexpression of MFN2, and of its yeast homolog Fzo1, led to apoptosis induction (Sugioka et al., 2004; Neuspiel et al., 2005). Further supporting a positive role of mitofusins in apoptosis, MFN2 protein levels are decreased in urinary bladder carcinoma tissues and its overexpression in a cellular model of the disease decreased cell viability via apoptosis induction (Jin et al., 2011). Similarly, in atherosclerosis or restenosis, MFN2 expression is downregulated. In vascular smooth muscle cells, overexpression of Mfn2 induces growth arrest (Guo et al., 2007). In fact, adenoviral expression of MFN2 showed an anti-tumor effect *in vitro* in a wide range of different cancer cell lines (Wu et al., 2008). MFN1 overexpression was also shown to induce apoptotic cell death of osteosarcoma cells, reducing malignancy, while the microRNA miR-19b, predicted to downregulate MFN1, had the opposite effect (Li X. et al., 2014). However, apoptosis is generally accompanied by mitochondrial fragmentation and, indeed, MFN2 was shown to be phosphorylated and proteasomally degraded upon genotoxic stress, which also induced apoptosis (Leboucher et al., 2012). Besides, *Mfn2* depletion in murine liver was shown to aggravate apoptosis provoked by bavachin, a flavonoid causing ER-stress, and *Mfn2* depletion in the hippocampus led to neuronal death (Yang et al., 2018; Han et al., 2020). Other than leading to mitofusins degradation, apoptosis might inactivate the fusogenic capacity of mitofusins, perhaps directly mediated by activated BAX/BAK oligomers (Karbowski et al., 2006). Consistently, when in its apoptotic conformation, BAX was proposed to inhibit Mfn2 activity (Hoppins et al., 2011). Moreover, BAK was reported to promote mitochondrial fragmentation during apoptosis by dissociating from Mfn2 and associating with Mfn1 (Brooks et al., 2007). Reciprocally, in healthy cells, BAX and BAK were proposed to promote mitochondrial fusion (Karbowski et al., 2006; Hoppins et al., 2011). Mechanistically, BAX and BAK seem to be required for Mfn2 assembly in fusion-prone complexes (Karbowski et al., 2006). Indeed, Mfn2 foci observed in wild type cells were impaired in the absence of these pro-apoptotic proteins, resulting in a more even mitochondrial distribution of Mfn2. Finally, BAX/BAK-loss induces mitochondrial fragmentation in similar extent to *Mfn2* knockout. Interestingly, mitofusins have also been linked with other types of cell death such as necroptosis, pyroptosis and ferroptosis (Xie et al., 2018; Bock and Tait, 2020). Necroptosis induction promoted ubiquitination and degradation of Mfn1, without affecting Mfn2 (Baker et al., 2014). Instead, upregulation of Mfn2 levels correlated with reduced pyroptosis in the liver, reverted by Oroxylin A, which allowed resisting to lipid deposition and ROS overproduction (Kai et al., 2020). In contrast, Mfn2 fusion activity was stimulated by the same BAX mutants that induced necrosis (Whelan et al., 2012; Karch et al., 2013) and hepatic knockdown of Mfn2 reduced ferroptosis, provoked by arsenite (Wei et al., 2020). In sum, the dual interplay between mitofusins and cell death points to multiple cross-talk effects that require future clarification.

## Mitophagy: A Pro-Survival or a Pro-Apoptotic Mechanism?

**FIGURE 2:**
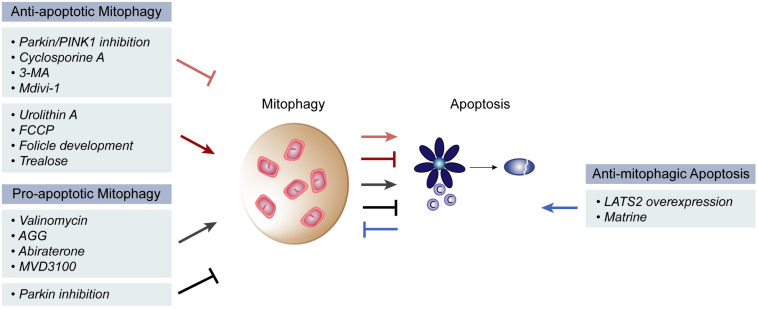
Pro- and anti- apoptotic roles of mitophagy. Mitophagy is reported to both induce and inhibit apoptosis in different physiological and pathological contexts. Inhibition of mitophagy via Parkin/PINK1 inhibition, Cyclosporine A, 3-MA or Mdivi-1 treatment has the ability to induce mitochondrial apoptosis, while induction of mitophagy that occurs during follicle development or upon urolithin A, FCCP or trealose treatment, has the opposite effect. On the other hand, pro-apoptotic roles of mitophagy were described upon mitophagy induction via valinomycin, AGG, abiraterone or MVD3100 treatment. Further, mitophagy suppression by parkin inhibition also inhibited apoptosis. Finally, an anti-mitophagic role of apoptosis could be observed upon overexpression of the large tumor suppressor gene 2 (LATS2) gene or by matrine treatment.

Mitophagy, autophagy and apoptosis–as major quality control mechanisms–are intimately related and often reported to affect each other (Maiuri et al., 2007; Kubli and Gustafsson, 2012; Anding and Baehrecke, 2015; Bloemberg and Quadrilatero, 2019). It is overall accepted that mitophagy prevents cell death, by clearing damaged and toxic mitochondria, thus constituting a pro-survival mechanism. However, pro-apoptotic and anti-tumor consequences of mitophagy were also reported (Figure 2).

### Anti-apoptotic Effects of Mitophagy

The majority of the findings showing a direct regulation of these two processes support the idea that mitophagy occurs as a pro-survival mechanism. Prevention of mitophagy, by Parkin and/or PINK1 inhibition, or by cyclosporine A or 3-MA treatment, induced CytC release and caspase activity, stimulating apoptosis (Tian et al., 2019; Kang et al., 2020; Li C. et al., 2020; Lin et al., 2020). Consistently, mitophagy induction via Urolithin A or FCCP treatment prevented apoptosis (Tian et al., 2019; Lin et al., 2020). Importantly, the inverse correlation between apoptosis and mitophagy was also observed under physiological conditions involving hypoxia. During follicle development, the follicle stimulating hormone is responsible for ensuring survival of porcine granulosa cells, despite their hypoxic environment (Li C. et al., 2020). In fact, the follicle stimulating hormone induced mitophagy, thereby protecting from hypoxia-induced apoptosis. The anti-apoptotic effects of mitophagy are also relevant in the context of liver, where an important role is attributed to mitofusins, as detailed in the next two chapters. For example, in murine hepatocytes, the impairment of mitophagy by downregulation of Parkin or PINK1, or by the knockout of both, was shown to significantly increase the hepatic apoptotic rate (Chen et al., 2019; Wang H. et al., 2019; Zheng et al., 2020). Similarly, in hepatocellular carcinoma HepG2 cells, treatment with Mdivi-1, a mitophagy inhibitor, led to induction of apoptosis (Kang et al., 2019). Inversely, interference with apoptosis also impacted on mitophagy. Apoptosis induction via matrine treatment, or by expression of the large tumor suppressor gene 2 (*LATS2*) suppressed mitophagy (Wei et al., 2018; Tian et al., 2019). Cell death was prevented by mitophagy activation, through HIF-1α-PINK1-Parkin, resolving hypoxic stress. General autophagy was also linked to apoptosis. Induction of autophagy with trealose delayed MOMP, while downregulation of autophagy components activated apoptosis, by increasing the levels of the p53-dependent apoptosis mediator (PUMA) (Thorburn et al., 2014). Mechanistically, autophagy inhibition caused accumulation of the transcription factor FOXO3a, increasing *PUMA* mRNA levels, thus sensitizing cells to apoptosis (Fitzwalter et al., 2018).

### Pro-apoptotic Effects of Mitophagy

A pro-apoptotic mechanism of mitophagy was also reported. Induction of mitophagy in cancer cells, by treatment with valinomycin, *Abrus* agglutinin (AGG), abiraterone or MDV3100 (anti-cancer drugs), led to an increase in apoptosis, facilitating cancer recovery (Panda et al., 2018; Ding et al., 2019; Han et al., 2019). The Bcl-2 family member BCL-B and the levels of phosphorylated Parkin appeared to be central in this response (Ding et al., 2019). Consistently, Parkin silencing decreased the death rate of hepatocellular carcinoma cells (Prieto-Domínguez et al., 2016). Further, a concomitant increase in mitophagy and apoptosis was observed upon *PUMA* overexpression or by treatment with the ribosome inhibitor AGG, which depended on BAX (Yee et al., 2010; Panda et al., 2018). Consistently, inhibition of mitophagy led to decreased caspase activity and apoptosis (Panda et al., 2018). Thus, synergistic induction of mitophagy and apoptosis, observed in glioblastomas cells or in lymph node prostate carcinoma cells (Panda et al., 2018; Han et al., 2019), appears to be relevant in suppressing cancer proliferation. However, given that anti-cancer drugs affect mitochondrial function, a causal pro-apoptotic role of mitophagy induction is still controversial.

Interestingly, beyond compensatory or synergistic effects, the ubiquitylation state of VDAC was shown to differentially affect either mitophagy or apoptosis. While polyubiquitylated VDAC1 is able to induce mitophagy, monoubiquitylated VDAC1 seems to exert a protective effect over apoptosis (Ham et al., 2020). However, the conditions by which VDAC gets mono or polyubiquitylated and the respective E3 ligases involved are still to be identified.

### Interaction Between Mitophagic and Apoptotic Components

The relationship between mitophagy and apoptosis is supported by evidence of physical interactions between several players of each process. First, the anti-apoptotic BCL-2 proteins Bcl-xL and MCL1 were shown to prevent mitophagy by physically interacting with Parkin, preventing its stable recruitment to mitochondria (Hollville et al., 2014; Yu et al., 2020). Second, the Bcl-2 pro-apoptotic family member BNIP3 is also a mitophagy receptor that physically interacts with LC3, via its LIR domain, promoting mitophagy. Interestingly, upon starvation, Beclin-1 participates in autophagosome formation, by associating with the hVps34/Class III PI3K complex, which localizes autophagy proteins to the autophagosome membrane. However, in nutrient rich conditions, the anti-apoptotic protein Bcl-2 interacts physically with the autophagic protein Beclin-1, preventing autophagy (Pattingre et al., 2005). By binding to Beclin-1, Bcl-2 impedes the formation of the hVps34/Class III PI3K complex, blocking autophagy. In contrast to this role of anti-apoptosis components in preventing autophagy via Beclin-1 sequestration, Beclin-1 was shown to induce apoptosis, by localizing to mitochondria (Wirawan et al., 2010). In fact, Beclin-1 is cleaved by caspases during apoptosis induction (Cho et al., 2009; Luo and Rubinsztein, 2010; Wirawan et al., 2010) and a Beclin-1 cleaved form localizes to mitochondria, sensitizing cells to apoptosis by enhancing CytC and HtrA2/Omi release (Wirawan et al., 2010). Similarly, after being cleaved by calpain, the autophagic protein Atg5 associated to the anti-apoptotic protein Bclx_1_, enhancing apoptosis (Yousefi et al., 2006).

## Interplay Between Mitochondrial Fusion, Mitophagy and Apoptosis

**FIGURE 3:**
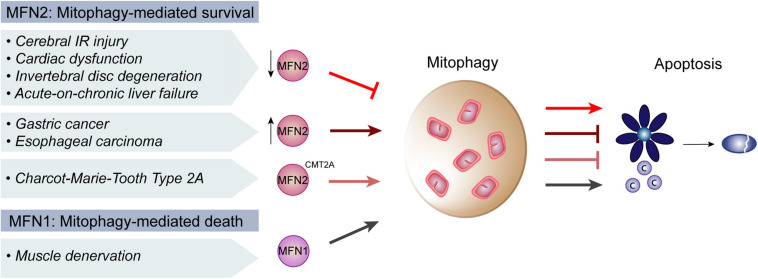
Regulation of mitophagy and apoptosis by mitofusins in disease. In cerebral ischemia-reperfusion (IR) injury and coronary heart disease, there is a presumable decrease of Mitofusin 2 (MFN2) levels, which repressed mitophagy and induced apoptosis. Similarly, in the context of intervertebral disc degeneration, acute-on-chronic liver failure and cardiac injury, decreased MFN2 levels were concomitant with decreased mitophagy and increased apoptosis. Consistently, digestive track cancer presented increased levels of MFN2, increased mitophagy and decreased apoptosis. Moreover, MFN2 mutations causative of the neuropathy Charcot-Marie-Tooth Type 2A were also reported to induce mitophagy and block apoptosis. In contrast, upon muscle denervation, low levels of Mitofusin 1 (MFN1) lead to an induction of mitophagy which promoted apoptosis, supporting an anti-apoptotic role of mitofusins.

Mitofusins–mainly MFN2 but recently also MFN1–have been broadly suggested as decision-making players in the interplay between mitophagy and apoptosis. Several pathological contexts affecting distinct physiological systems, such as heart, liver and brain, have implied a role of mitofusins. Generally, mitofusins are downregulated in disease states, being pathophysiology improved by restoring mitofusin’s levels, thus highlighting a broad therapeutic potential of these proteins (Figure 3). Regarding Mfn1, peripheral nerve injury and consequent muscular denervation upregulated the miRNA 142a-5p, which was bioinformatically predicted to target *Mfn1* (Yang et al., 2020). Consistently, denervated muscles presented decreased levels of Mfn1. Moreover, a miRNA-142a-5p mimic led to downregulation of *Mfn1* and to increased mitophagy and apoptosis of skeletal muscle cells, which were rescued by a miRNA-142a-5p-inhibitor. Experiments performed *in vivo*, in mice sciatic nerve transection models, confirmed these observations, pointing to an important role of Mfn1 in protecting from mitophagy and apoptosis (Yang et al., 2020).

Decreased levels of MFN2 inhibited mitophagy and increased apoptosis. While rather exacerbating damage caused by stress in healthy cells, compromising mitophagy and MFN2 lead to better disease prognoses in cancer. For example, in gastric cancer, increased levels of Yes-associated protein (YAP) contribute to cellular proliferation and metastasis (Yan H. et al., 2018). Knockdown of *YAP* inhibited Sirtuin 1 (SIRT1) activity, consequently decreasing *MFN2* expression and mitophagy. This increased apoptosis and oxidative stress, preventing migration of the cancer cells. Upregulation of mitophagy in YAP-deficient cells, with FCCP or by reactivation of SIRT1, reversed apoptosis induction. This substantiated the importance of MFN2 and mitophagy inhibition in gastric cancer treatment (Yan H. et al., 2018). Similarly, upon treatment of esophageal squamous cell carcinoma with the cytokine IL-24, drug-resistance responses were associated with increased mitophagy (Zhang J. et al., 2019). Drug-resistance also correlated with decreased levels of macrophage stimulating factor 1 (MST1). Overexpression of *MST1* inhibited ERK activity, decreasing MFN2 levels and consequently preventing mitophagy. This was accompanied by better anti-cancer efficacy of IL-24. Importantly, independent silencing of *MFN2* or chemical inhibition of ERK led to the same mitophagic outcome (Zhang J. et al., 2019). In sum, the pro-survival role of MFN2 and mitophagy in cancer cells suggest it as a potential target for inhibition in oncogenic treatments.

The role ERK-MFN2 in mitophagy was also described in cerebral ischemia-reperfusion (IR) injury (Zhang and Yu, 2018). Here, decreased mitophagy and increased apoptosis are associated with brain damage. The protein Nr4a1 was upregulated upon IR injury, which induced brain damage by increasing apoptosis and by inhibiting mitophagy. Mitophagic rescue and apoptotic inhibition, caused by *Nr4a1* deletion, were lost in absence of *Mfn2*, pointing to a dependence of Nr4a1on Mfn2 for IR injury. IR injury also repressed ERK and CREB phosphorylation, again reverted by Nr4a1 loss, implying the MAPK-ERK-CREB signaling pathway in this response. Furthermore, in Nr4a1-deficient cells, blockage of the MAPK-ERK-CREB signaling pathway decreased the levels of Mfn2 and no longer allowed apoptotic inhibition. In sum, this study points to an important role of Mfn2 and of the MAPK-ERK-CREB pathway in the brain, by controlling mitophagy and apoptosis upon IR injury (Zhang and Yu, 2018). The importance of MFN2 in the interplay between mitophagy and apoptosis was confirmed in the context of coronary heart disease, however instead involving the AMPK-CREB pathway (Li P. et al., 2020). Inflammation in human umbilical vein endothelial cells, caused by oxidized low-density lipoprotein (ox-LDL), induced the phosphatase and tensin homolog (PTEN), decreased mitophagy and increased apoptotic cell death. PTEN downregulation reversed these phenotypes, being the rescue dependent on the presence of MFN2. Furthermore, ox-LDL treatment disrupted AMPK activity and decreased CREB phosphorylation, being both phenotypes rescued by PTEN deletion. Finally, AMPK inhibition led to concomitant reduction of *MFN2* expression, pointing to a role of the AMPK-CREB pathway and of MFN2-induced mitophagy in preventing apoptosis and heart injury (Li P. et al., 2020). Also in heart, Mfn2 was implicated in the regulation of cardiomyocyte cell death mediated by the kinase Lats2 (Tian et al., 2019). *Lats2* overexpression resulted in decreased levels of Mfn2, reduced mitophagy and increased cardiomyocyte apoptosis. Consistent with the usual association of cardiomyocyte death with hypoxia, the levels of peroxiredoxin 3 (Prx3), a reactive oxygen species scavenger, were decreased upon *Lats2* overexpression. In *Lats2* overexpression conditions, Prx3 re-expression rescued Mfn2 levels and mitophagy, pointing to an important role of Prx3 (Tian et al., 2019). Thus, it would be interesting to test if reactivation of Prx3-Mfn2-mitophagy reverses Lats2-induced cardiomyocyte death. In sum, stress conditions seem to cause a decrease in mitophagy, caused by Mfn2 depletion through the downregulation of associated signaling pathways.

Mitofusin 2 downregulation, causing decreased mitophagy or autophagy, was also observed in cardiac injury mimicked by angiotensin II (Xiong et al., 2019), in intervertebral disc degeneration (IVDD) (Chen et al., 2020) and in acute-on-chronic liver failure (ACLF) (Xue et al., 2019a,b). Common to these reports, low levels of MFN2/Mfn2 and concomitant decrease in autophagy/mitophagy were accompanied by apoptosis induction. Importantly, MFN2 re-expression rescued autophagy/mitophagy levels and reversed apoptosis. Reinforcing the great dependence of these two cellular processes on each other, Chen et al. (2020) further showed that such protective effects of MFN2 over apoptosis are dependent on the autophagic flux during IVDD (Chen et al., 2020). In sum, these reports correlate disease to decreased MFN2, to decreased mitophagy and to induction of apoptosis. Inversely, in CMT2A neuropathy models, where MFN2 itself is present in a mutated form, mitophagy was induced and apoptosis was reduced (Rizzo et al., 2016). Indeed, using motor neurons differentiated from CMT2A patient fibroblasts-derived iPSCs as a cellular model of the disease, the authors observed increased autophagic clearance of mitochondria and decreased levels of BAX, caspase 8 and caspase 3 cleavage as well as increased levels of the anti-apoptotic BCL-2 protein (Rizzo et al., 2016). In conclusion, mitofusins, especially MFN2, regulate cell death by mediating mitophagy (Figure 3).

## MFN2 and Mitophagy in Non-Alcoholic Fatty Liver Disease (NAFLD)

**FIGURE 4:**
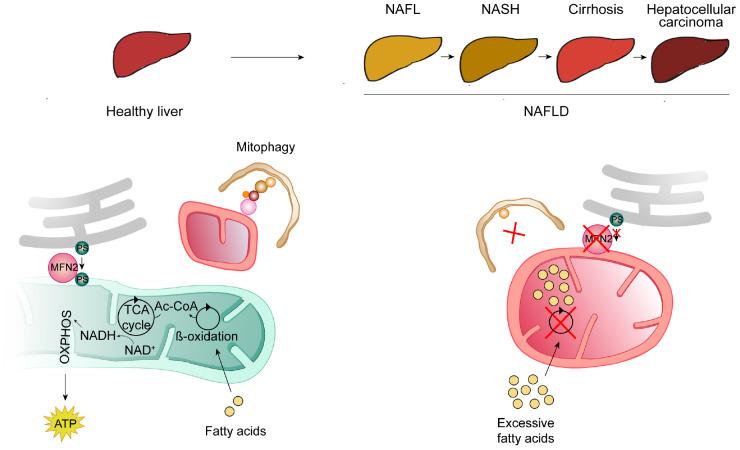
Mitochondrial contribution for the development of non-alcoholic fatty liver disease (NAFLD). NAFLD consists of several heterogeneous clinical manifestations, usually organized in a development-stepwise-spectrum. The first step (NAFL) consists of the pathological accumulation of fat in the hepatocytes, due to excessive lipid intake and insufficient adipose storage. NAFL can evolve to further steatosis, accompanied by inflammation and scarring [(non-alcoholic steatohepatitis (NASH)], followed by cirrhosis and, ultimately, hepatocellular carcinoma. Mitochondrial dysfunction, also caused by low levels of MFN2, contributes to the development of NAFLD. In a healthy liver (left panel), free fatty acids are metabolized by β-oxidation into Acetyl-CoA (Ac-CoA), which can then enter the citric acid (TCA) cycle, from which NADH is produced and further used by the oxidative phosphorylation chain (OXPHOS) to produce energy in the form of ATP. These healthy mitochondria are kept in check by mitophagy (for details see Figure 1). Also in a healthy situation (left panel), MFN2 was proposed to serve as a tether between mitochondria and ER and thereby to also bind to and promote the transfer of phosphatidylserine (PS) from the ER to mitochondria. In a situation of excessive lipid intake and NAFLD development (right panel), mitophagy is impaired, leading to the accumulation of dysfunctional mitochondria, unable to metabolize fatty acids, which accumulate in hepatic mitochondria and lead to steatosis. Furthermore, the absence of MFN2 impairs PS transfer to mitochondria, affecting further lipid biosynthesis, consequently leading to ER stress in hepatocytes.

A high-incidence disease where a strong interplay between mitophagy and apoptosis can be found is NAFLD, which affects around 25% of the world’s population and is the most common hepatic disease in the western countries (Kumar et al., 2020). It is a step-wise liver disease characterized by a spectrum of heterogeneous clinical manifestations. It is initiated by the excessive accumulation of fat in the liver, named steatosis. Although patients can exhibit steatosis without further complications, in many cases steatosis evolves to hepatic inflammation, fibrotic restructuring, cirrhosis, hepatocellular carcinoma and ultimately liver failure (Parthasarathy et al., 2020; Figure 4). Other disorders can constitute risk factors for NAFLD development, such as type 2 diabetes mellitus, obesity, metabolic syndrome, cardiovascular disease, and chronic kidney disease (VanWagner and Rinella, 2017; Li A. A. et al., 2020). Interestingly, a bidirectional link was observed for some of these, since NAFLD is a risk factor for the development of cardiovascular disease, atherosclerosis and chronic kidney disease (VanWagner and Rinella, 2017).

### NAFLD and Mitochondria

There are numerous scientific reports showing an induction of apoptosis in the progression of NAFLD (Feldstein et al., 2003; Ferreira et al., 2011; Li C. P. et al., 2014; Alkhouri et al., 2015; Gonçalves et al., 2015; Kanda et al., 2018). Enhanced apoptosis in NAFLD hepatocytes correlates with activation of caspases (Feldstein et al., 2003; Ferreira et al., 2011) and both FAS -an apoptosis signal transduction factor- and its ligand -FAS-L- were found upregulated in NAFLD (Feldstein et al., 2003; Li C. P. et al., 2014; Alkhouri et al., 2015). NAFLD and mitochondria are also intimately related, being mitochondrial dysfunction and structural changes hallmarks of this hepatic disease. Upon high lipid intake, when adipocyte storage is no longer sufficient, hepatocytes uptake, store and metabolize lipids as well (Singh et al., 2009). In the liver, free fatty acids can then either undergo β-oxidation within mitochondria or be esterified into triglycerides (Figure 3). In early stages of NAFLD, mitochondria upregulate their activity, resolving lipid overload. However, continuous excess of lipid intake can impair mitochondrial function (Flores-Toro et al., 2016; Lee et al., 2019). Both NAFLD patient biopsies and animal models frequently present functional impairment of β-oxidation and of the respiratory chain and altered mitochondrial morphology (Paradies et al., 2014). Hence, a failure in mitochondrial quality control by mitophagy, and the subsequent accumulation of dysfunctional mitochondria, can contribute to the pathological accumulation of fatty acids in the liver (Figure 3). Consistently, reduced levels of PINK1/Parkin and thus reduced mitophagy was found in a NAFLD mouse model, upon high-fat diet (Gonçalves et al., 2015). Moreover, PINK1/Parkin-dependent induction of mitophagy, by the administration of quercetin–a plant flavonoid–rescued hepatic steatosis, both in *in vitro* and *in vivo* models of NAFLD (Liu et al., 2018). Similar beneficial results were obtained when using low doses of sorafenib, an anti-tumor drug used in hepatocellular carcinoma treatment (Jian et al., 2020). Sorafenib prevented progression of non-alcoholic steatohepatitis (NASH, the primary stage of NAFLD), in mice and monkeys, by inducing mitochondrial uncoupling, associated with activation of mitophagy. Importantly, endurance training reversed the status of several high fat-diet markers, leading to a decrease in Ca^2+^-dependent mitochondrial swelling, in both BAX and caspase 8 and 9 activities and also a rescue of PINK1/Parkin levels and of the mitochondrial biogenesis markers TFAM and PGC-1α (Gonçalves et al., 2015). Thus, exercise can help mitigate over-nutrition damage in mitochondrial functions and reverse apoptosis induction, preventing the development of NAFLD (Gonçalves et al., 2015).

Mechanistically, it is not well understood how mitophagy is impaired in NAFLD. However, recent studies under high-fat diet brought some insights onto why lack of mitophagy and enhanced apoptosis disrupt liver functionality. Notably, these NAFLD mouse models revealed increased levels of nuclear receptor subfamily 4 group A member 1 (Nr4a1), which repressed Bnip3 (Zhou et al., 2017). BNIP3 protein levels are regulated by nutrient availability, being reduced in fasting conditions. Moreover, although first annotated as a cell-death regulatory factor, several studies reported roles of Bnip3 in mitochondrial dysfunction and in mitophagy, being recognized as a mitophagy receptor, which can also assist in cell survival (Gao et al., 2020). Absence of Bnip3 as well as high-fat diet lead to mitophagy inhibition along with increased cell death (Zhou et al., 2017). *BNIP3* deleted mice exhibit lipid accumulation and steatosis, *in vitro* and *in vivo*, consistent with increased hepatocyte lipogenesis and decreased β-oxidation of fatty acids (Glick et al., 2012). Furthermore, *BNIP3* null murine hepatocytes present an increase in mitochondrial mass, associated with a decline in mitochondrial function, consistent with defective mitophagy (Glick et al., 2012). In NAFLD mouse models, loss of Nr4a1 relieved Bnip3 repression and re-activated mitophagy. Importantly, it also mitigated NAFLD-phenotypes, namely mice body and liver weight, hepatocyte vacuolation, hepatic lipid accumulation, steatosis and hepatic fibrosis (Zhou et al., 2017). Melatonine, used as anti-oxidant, anti-inflammatory and anti-obesogenic drug, also attenuated NAFLD-associated phenotypes induced by high-fat diet, suggesting it could possibly be used as a supplement for the treatment of NAFLD (Zhou et al., 2017).

High-fat stress, both *in vivo* and *in vitro*, also led to upregulation of the growth suppressor Mst1 (Zhou T. et al., 2019). *Mst1* deletion rescued the metabolic NAFLD signature phenotypes, namely body weight, blood glucose levels, triglycerides levels, total cholesterol and levels of lipid metabolism enzymes (Zhou T. et al., 2019). Equally, *Mst1* deletion suppressed abnormal liver structure, liver weight, hepatocytes size and liver fibrosis. The effects of hepatic injury reversion attained by *Mst1* deletion were further attributed to a reduction in oxidative stress and inflammation response. Consistently, *Mst1* deletion ameliorated hepatic steatosis (Jeong et al., 2018). Finally, downregulation of *Mst1* also rescued mitochondrial potential and prevented mPTP opening, CytC release and caspase activation (Zhou T. et al., 2019). Moreover, rescue of apoptosis was dependent on Parkin (Zhou T. et al., 2019), pointing to a strong correlation between apoptosis and mitophagy in the development of NAFLD. Besides NAFLD, MST1/Mst1 also repressed mitophagy during cardiac ischemia-reperfusion injury and colorectal cancer (Li et al., 2018; Yu et al., 2019). Thus, although still to be mechanistically defined, Mst1 appears to generally cause inhibition of mitophagy. In conclusion, Mst1 and Nr4a1 inhibition restored mitophagy and reduced cell death, alleviating high-fat stress, of clinical relevance in the context of NAFLD.

Despite most studies reporting an inhibition of mitophagy at the basis of NAFLD development, mitophagy induction along with hepatocytes’ apoptosis was also observed (Pang et al., 2018). Upon oleic acid treatment, shown to induce NASH in HepG2 cells (Cui et al., 2010), reticulophagy is induced, preventing the death of hepatocytes. However, continuous lipid intake induced mitophagy, accompanied by an increase in hepatocytes’ apoptosis, which could be prevented by PINK1 downregulation (Pang et al., 2018). Nevertheless, why excessive lipid intake caused mitophagy induction, rather than the mostly described mitophagy failure and mitochondrial dysfunction, is still unclear.

### NAFLD and MFN2

Morphological alterations of mitochondria, characterized by very enlarged organelles, have been broadly observed upon liver dysfunctions, constituting a hallmark of NAFLD and alcoholic liver injury (Caldwell et al., 1999; Wakabayashi, 2002; Noureddin et al., 2013; Lotowska et al., 2014; Neuman et al., 2014; Kleiner and Makhlouf, 2016; Yamada et al., 2018). Importantly, two recent studies provided causal implications of mitochondrial shape and dynamics proteins in NAFLD (Yamada et al., 2018; Hernández-Alvarez et al., 2019). First, blocking either fusion or fission was harmful in hepatocytes, suggesting that extreme mitochondrial lengths–fragmented or hypertubular–affected liver functionality (Yamada et al., 2018). Consistently, mitochondrial stasis, created by simultaneously blocking mitochondrial division (Drp1 knockout) and fusion (Opa1 knockout) re-established mitochondrial size and mitigated pathological markers. Moreover, re-establishment of mitochondrial size rescued mitophagy and liver damage (Yamada et al., 2018). Second, MFN2 was proposed to directly assist in phosphatidylserine (PS) transfer from the ER into mitochondria, thus protecting against NAFLD (Hernández-Alvarez et al., 2019; Figure 3). In this study, both liver biopsies from NASH patients and mouse models of steatosis showed reduced Mfn2 levels. Consistently, murine liver-specific ablation of *Mfn2* led to abnormal lipid metabolism, chronic hepatic inflammation, apoptosis, fibrosis and liver cancer (Hernández-Alvarez et al., 2019). Mechanistically, *Mfn2* ablation in the liver was accompanied by a decrease in the levels of phosphatidylserine synthase 1 and 2. Moreover, Mfn2 was able to selectively bind PS, indicating a direct role of MFN2 in phospholipid regulation (Hernández-Alvarez et al., 2019). The authors therefore suggested that MFN2 hepatic deficiency leads to inefficient PS transfer from ER to mitochondria. Then, the subsequent inability to synthetize other phospholipids, such as phosphatidylethanolamine, causes ER stress (Hernández-Alvarez et al., 2019). Indeed, ER stress was shown to induce NAFLD-related phenotypes (DeZwaan-McCabe et al., 2013). Finally, re-expression of Mfn2 was able to restore normal liver metabolism, suggesting therapeutic potential (Hernández-Alvarez et al., 2019). In conclusion, MFN2 downregulation, mitophagy defects and pathological accumulation of lipids are determinant in the development of NAFLD disease.

## Concluding Remarks

**FIGURE 5:**
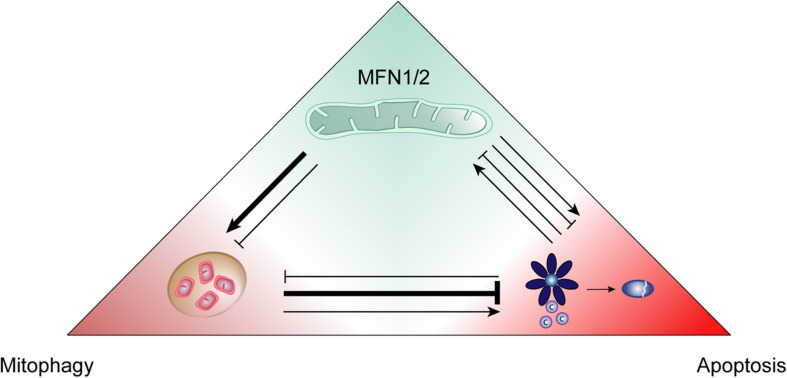
Positive and negative interactions between mitofusins, mitophagy and apoptosis. The quality control processes of mitophagy and apoptosis can positively and negatively regulate each other and, in turn, this interplay can be itself influenced by mitofusins (MFN1/2). Mitophagy mostly behaves as an anti-apoptotic mechanism, promoting cellular survival. However, it can also contribute to cell death by apoptosis. Mitofusins add another layer of regulation since they are known to promote both mitophagy and apoptosis. Nevertheless, these proteins can also be found to negatively impact mitophagy induction.

The crosstalk between the major surveillance mechanisms of mitophagy and apoptosis amplifies the cellular capacity to ensure cellular homeostasis. Recent studies placed mitofusins at the cross-roads of these two quality control processes (Figure 5). The identification of mitofusins’ manifold central functions, as described here, provides the basis for future mechanistic understanding of this interdependence. Future studies will certainly shed light on the molecular details involving mitofusins in mitophagy, apoptosis and their interplay. As such, mitofusins hold promise for developing targeted therapeutic approaches.

## Author Contributions

Both authors conceived the study and the figures. MJ wrote the manuscript and drew the figures. ME-H coordinated the study. Both authors contributed to the article and approved the submitted version.

## Conflict of Interest

The authors declare that the research was conducted in the absence of any commercial or financial relationships that could be construed as a potential conflict of interest.
